# Staphylococcus Lugdunensis Endocarditis and Cerebrovascular Accident: A Systemic Review of Risk Factors and Clinical outcome

**DOI:** 10.7759/cureus.2469

**Published:** 2018-04-12

**Authors:** Htoo Kyaw, Felix Raju, Atif Z. Shaikh, Aung Naing Lin, Aye T. Lin, Joseph Abboud, Sarath Reddy

**Affiliations:** 1 Division of Cardiology, Brooklyn Hospital Center/Mount Sinai Hospital, New York, USA; 2 Internal Medicine, Brooklyn Hospital Center/Mount Sinai Hospital, New York, USA; 3 Department of Continuing Care, The Southern California Permanente Medical Group, Bakersfield, USA

**Keywords:** staphylococcus lugdunensis, endocarditis, cerebrovascular accident, cardiology, infectious endocarditis

## Abstract

Infective endocarditis (IE) secondary to Staphylococcus lugdunensis has been increasingly recognized since 1988. IE-related thromboembolism represents an associated complication of the disease and carries a dismal prognosis. However, the incidence of cerebrovascular accident secondary to S. lugdunensis IE is relatively uncommon and its treatment has not been clearly elucidated yet.

We performed an extensive literature search using Pubmed, Medline, Scopus, and Google Scholar to identify the articles using the following keywords: ‘Staphylococcus lugdunensis’, ‘infective endocarditis’, ‘stroke’, and ‘cerebrovascular accident.’ Patient characteristics, risk factors, severity of neurological deficit, echocardiographic findings, medical management, required surgical intervention, complications and mortality rate were reviewed in detail.

Eighteen cases (mean age of 47.8 years, 55% male) from 17 publications with S. lugdunensis-related cerebrovascular accident (CVA) were identified. Of these, 16 (87%) cases were left-sided endocarditis and 10 (61%) cases experienced right-sided neurological deficit. The source of infection was documented in eight cases (50%) in which four cases (50%) were related to groin-related procedures and the mitral valve (52.5%) was mostly infected followed by aortic valve (37%). Surgical valve replacement was done in 61% of patients and overall mortality rate was 22%.

S. lugdunensis endocarditis is associated with high mortality and morbidity, including a higher prevalence of CVA. Early disease identification with aggressive intervention is crucial for better outcomes.

## Introduction and background

Infective endocarditis (IE) has long been recognized and treated over three centuries starting from 1674 when Lazare Riviere first noticed IE as a disease [1]. The overall annual incidence of IE varies from 3 to 7 per 100,000 person-years in the global population study while Bor et al. reported that approximately 40,000 IE cases/year with a 14.5% mortality rate occurred in the USA [2,3]. IE can be caused by various pathogens including bacteria and fungus although the former is more common. Due to frequent invasive procedures such as cardiac catheterization, defibrillator and central line placement, Staphylococcus aureus has become the most causal organism followed by Streptococcus species with an estimated incidence of 49.3% and 24.7%, respectively [2].

Coagulase-negative Staphylococci (CoNS) is known to be a nonpathogenic commensal in individuals with normal immune response. However, this concept changed when Staphylococcus lugdunensis, a member of the CoNS group, was first identified as a causal pathogen of IE in 1988 by Freney et al. [4]. Although most CoNS tend to have more latent pathogenicity, S. lugdunensis is an exception with a higher degree of virulence similar to Staphylococcus aureus. S. lugdunensis often requires immediate and aggressive treatment including surgical intervention. Embolic complications related to IE represent a more severe endpoint of the disease including S. lugdunensis endocarditis-induced embolic stroke. The question regarding how and when to start treatment remains unclear as no definitive management plan is described in the guidelines.

This review article aims to evaluate the clinical presentation, characteristics and risk factors, as well as the necessity of surgical treatment in cerebrovascular accident (CVA) due to S. lugdunensis endocarditis.

## Review

Materials and methods

**Figure 1:**
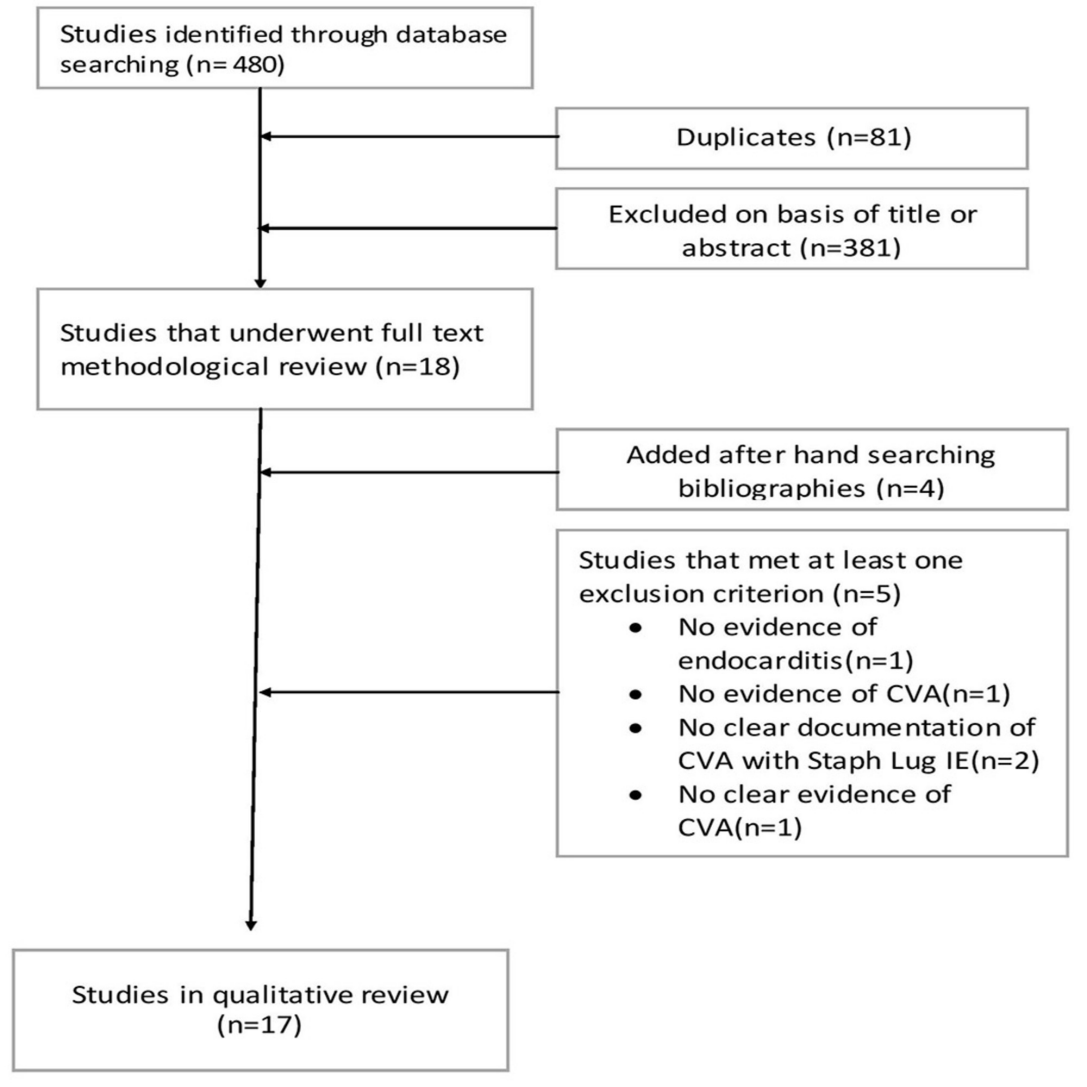
Literature search and selection. CVA: Cerebrovascular accident.

We performed an extensive literature search using Pubmed, Medline, Scopus, and Google Scholar to identify peer-reviewed original research, review articles, case reports and series using ‘Staphylococcus lugdunensis’, ‘infective endocarditis’, ‘stroke’, and ‘cerebrovascular accident’. The search period included all the articles published until January 2017. Search result yielded mostly case reports and case series. Patient characteristics, risk factors, echocardiographic findings, vegetation size, required surgical intervention and its complications, and mortality rate were reviewed in detail (Figure 1).

Results

A total of 17 original published articles were considered appropriate for inclusion in our review article (Figure 1). Out of these 17 studies, 16 were case reports from Italy [5,6], Switzerland [7], USA [8-11], UK [12,13], Morocco [14], Taiwan [15,16], Saudi Arabia [17], Spain [18,19], and Norway [20] while one case series was from Greece [21]. Koh et al. had reported the first case of S. lugdunensis endocarditis-associated CVA from London, UK in 1996 [12]. A total of 18 cases from 17 articles were included in the review article. All cases are summarized in Table 1 and Table 2.

**Table 1:** Baseline characteristics and demographic information. IE: Infective endocarditis; IV: Intravenous; TTE: Trans-thoracic echocardiogram; URI: Upper respiratory tract infection; LV: Left ventricle; PPM: Permanent pacemaker.

First author (year) [Ref. No.]	Total patients	Age (Years)	Sex (M/F)	IV Drug abuse history	Previous IE history	Portal of entry	Location of IE	Type of valve	Vegetation TTE (Y/N)
Htoo Kyaw (2016) [11]	1	63	1/0	Not-mentioned	None	Unknown	Mitral valve	Native mitral valve	Y
Hossein Schandiz (2015) [20]	1	56	1/0	Not-mentioned	None	Vasectomy	Mitral valve	Native mitral valve	N (autopsy finding)
Manova David (2015) [10]	1	36	1/0	Not-mentioned	None	Vasectomy	Mitral valve	Native mitral valve	Y
Jose Kuzhively (2014) [9]	1	61	1/0	Polysubstance abuse	None	Spinal fracture fixation	Mitral valve anterior leaflet	Native mitral valve	Y
Wei-Chi Tsai (2013) [15]	1	48	1/0	Not-mentioned	None	Femoral catheterization	Aortic valve	Native aortic valve	Y
Federico Pacei (2013) [6]	1	77	1/0	Not-mentioned	None	Mechanical valve replacement for AS and CABG	Mechanical aortic valve	Mechanical aortic valve	Y
Rosaria Pecoraro (2012) [5]	1	18	0/1	Not-mentioned	None	Not-mentioned	Anterior mitral valve leaflets	Native mitral valve	Y
Kalliopi-Stavroula Chatzigeorgiou (2010) [21]	2	67.5	0/2	Not-mentioned	None	Not-mentioned	LV outflow/PPM lead with Tricuspid valve	Prostatic aortic valve / native tricuspid valve with PPM	Y
Asmaa Tamdy (2010) [14]	1	17	0/1	Not-mentioned	None	Not-mentioned	Mitral valve	Native mitral valve	Y
Yeun Tarl Fresner Ng Jao (2010) [16]	1	44	1/0	Not-mentioned	None	Pneumonia	Mitral and aortic valve	Native mitral and aortic valve	Y
Khaled E. Alebrahim (2007) [17]	1	13	1/0	Not-mentioned	None	Not-mentioned	Mitral valve	Native mitral valve	Y
S. Gianella (2006) [7]	1	49	0/1	Not-mentioned	None	Not-mentioned	Bicuspid aortic valve	Native bicuspid aortic valve	Y
M Rodríguez-Gascón (2003) [18]	1	77	0/1	Not-mentioned	None	Valvular surgery/URI	Mitral valve	Native mitral valve	Y
A. Sánchez (2000) [19]	1	71	0/1	Not-mentioned	None	Not-mentioned	Aortic valve	Native aortic valve	Y
Susan J. Burgert (1999) [8]	1	33	1/0	Not-mentioned	None	Not-mentioned	Bicuspid aortic valve	Native bicuspid aortic valve	Y
T. W. Koh (1996) [12]	1	52	0/1	Not-mentioned	None	Not-mentioned	Mitral valve	Native mitral valve	Y
B Walsh and JP Mounsey (1990) [13]	1	32	1/0	Not-mentioned	None	Vasectomy	Aortic valve	Native aortic valve	N

**Table 2:** Summary of infective endocarditis treatment and outcome. CVA: Cerebrovascular accident; LVOT: Left ventricular outflow tract; PPM: Permanent pacemaker.

First author (year) [Ref. No.]	Vegetation size on echocardiography	CVA severity	Medical treatment (Y/N)	Penicillin resistance (Y/N)	Surgical intervention (Y/N)	Required valve replacement	Outcome
Htoo Kyaw (2016) [11]	Vegetation on anterior and posterior mitral valve leaflets	Aphasia and right-sided hemiplegia	Y	N	Y	Y	Recovered
Hossein Schandiz (2015)[20]	Massive vegetation on mitral valve	Not mentioned: presented as syncope	N	Y	N (planned)	N	Died before surgery
Manova David (2015) [10]	7 mm on anterior leaflets and 15 mm on posterior leaflet of mitral valve	Dysarthria and left-sided hemiparesis	Y	N	Y	Y	Recovered
Jose Kuzhively (2014) [9]	15.5 x 11.1 mm on the anterior leaflet of the mitral valve	Right hemiplegia and facial palsy	Y	N	N	N	Recovered
Wei-Chi Tsai (2013) [15]	Aortic valve vegetation with perivalvular abscess	Right internuclear ophthalmoplegia	Y	N	N (planned)	N	Died before surgery
Federico Pacei (2013) [6]	Not mentioned	Quadriplegia	Y	N	N	N	Recovered
Rosaria Pecoraro (2012) [5]	10.3 x 16.6 mm on anterior mitral valve leaflet	Right leg weakness	Y	N	Y	Y	Recovered
Kalliopi-Stavroula Chatzigeorgiou (2010)[21]	18 x 22 mm at LVOT/7 x 16 mm on PPM leads and small vegetation on tricuspid valve	Dysarthria and facial nerve paresis	Y/Y	N/N	Y/N (only removal of PPM wires)	N/N	Recovered (died 5 years later with calciphylaxis)/Recovered
Asmaa Tamdy (2010)[14]	17 x 5 mm on the anterior mitral valve with small vegetation on both leaflets of mitral valve	Right hemiplegia and aphasia	Y	N	N (planned)	N (planned)	Died before surgery
Yeun Tarl Fresner Ng Jao (2010) [16]	Not mentioned	Right hemiparesis	Y	N	Y	Y	Died
Khaled E. Alebrahim (2007) [17]	7 x 8 mm on anterior and 3 x 6 mm posterior leaflets of mitral valve	Right hemiparesis and aphasia	Y	N	Y	Y	Recovered
S. Gianella (2006) [7]	12 x 4 mm on the bicuspid aortic valve	Right hemiparesis and aphasia	Y	N	Y	Y	Recovered
M Rodríguez-Gascón (2003) [18]	3 x 3 cm vegetation on mitral valve	Left hemiparesis	Y	N	N	N	Recovered
A. Sánchez (2000) [19]	Not mentioned	Generalized weakness	Y	N	Y	Y	Recovered
Susan J. Burgert (1999) [8]	5 mm perforation with multiple vegetations on aortic valve	Dysarthria, aphasia and right hemiparesis	Y	N	Y	Y	Recovered
T. W. Koh (1996) [12]	Vegetation on posterior leaflets of the mitral valve	Dysphasia and right facial weakness	Y	N	Y	Y	Recovered
B Walsh and JP Mounsey ( 1990) [13]	Not mentioned	Right-hand weakness	Y	N	Y	Y	Recovered

Demographics

Most of the reported cases were from European countries and only two cases were from China. Four reported cases were from the USA and very limited published data regarding S. lugdunensis endocarditis-induced CVA has been noted in the past literature. Of these 18 cases, 10 were men and eight were women with mean age of 47.9 years (range 13-77 years).

Risk factors, patients’ characteristics and clinical spectrum

The risk factors associated with Staphylococcus lugdunensis are intravenous drug abuse, previous history of infective endocarditis, and surgical intervention in the groin area. Among patients with a documented source of infection, four cases (50%) were noted to have either vasectomy or femoral catheterization. No previous history of IE was reported in any of the cases.

Left-sided IE is the most common site involved, accounting for 94.7% of all total cases in which mitral valve, aortic valve, or both amounted to 52.5%, 37%, and 5.2%, respectively (Table 3). Foreign body-related IE comprised three cases including one case with pacemaker-related endocarditis and two cases of prosthetic valve IE. Sixteen individuals (88.88%) were found to have vegetations on echocardiogram with the largest measuring 3 cm x 3 cm in diameter on the mitral valve. The neurological deficit was mostly located in the right side (61%) with facial palsy in 17% and left-sided hemiparesis in 11% (Table 2).

**Table 3:** Characteristic, location of infected valves, and outcome of Staphylococcus lugdunensis infective endocarditis. LVOT: Left ventricular outflow tract.

	Total cases, N (%)
Type of infected valve	
Mitral valve, aortic valve, tricuspid valve, pulmonary valve, LVOT	10 (52.5%), 7 (37%), 1 (5.2%), 01 (5.2%)
Location of vegetation	
Left sided, right sided	17 (94.4%), 1 (5.5%)
Valve replacement	
Yes, No	11 (61.1%), 7 (39%)
Outcome	
Recovered, died	14 (78%), 4 (22%)

Investigation, medical and surgical treatment

Blood culture remains the gold standard to diagnose this type of bacteria and it should be in the differential whenever coagulase-negative Staphylococcus growth is present. Once S. lugdunensis is isolated, early antibiotic therapy and surgical intervention should be considered.

Antibiotic sensitivity testing showed methicillin susceptibility in most of the cases except one case (5.5%) which had penicillin resistance and was consistent with the previous reported literature. In studies by Anguera et al. and Hellbacher et al., S. lugdunensis was susceptible to penicillin with a rate of 84% and 85%, respectively [22,23]. Most of the cases were treated with sensitivity-guided medical therapy but 12 cases (66.6%) were eventually required to undergo surgical treatment. This particular finding is relatively higher than other pathogens including S. aureus.

Anguera et al. had reported a four-year study of IE in which surgical intervention was performed in Staphylococcus aureus IE (~37%), Staphylococcus epidermidis (~60%), and Staphylococcus lugdunensis (~70%) [22]. Another national study done by Ferreiros et al. and Krcmery et al. showed that surgical treatment with valve replacement was done in 26-42% of IE caused by all pathogens [24,25]. Despite S. lugdunensis IE-related CVA being mostly methicillin/penicillin sensitive, it is less likely to be eradicated by medical therapy alone and often requires surgical intervention.

Perhaps extrapolating from our experience in treating CVA associated with S. Lugdunensis IE, early surgical treatment was often required to have a better outcome. However, the latest 2015 AHA guideline has failed to mention any specific recommendation for this disease entity [26]. Thus, clinicians will have to make a decision based on their clinical experiences which could create a heterogeneity in disease management.

Prognosis

Unlike other organisms, S. lugdunensis is characterized by an insidious clinical course resulting in a higher mortality. The mortality rate was 22% (four out of 18 cases) but only one case out of 11 cases that underwent surgical intervention expired compared to those with medical treatment (8.3% vs 37.5%) (Table 3).

Anguera et al. disclosed that the changes in mortality rate before and after 1995 were primarily due to earlier recognition of S. lugdunensis with more aggressive management including surgical intervention [22]. According to the two studies done in 1993 and 2003, the mortality rate was 16% due to CoNS IE as well as 22.4% with S. aureus IE, which had a reduced mortality rate than our review [27,28]. In a 2010 review article done by Liu et al., among 68 patients with S. lugdunensis infective endocarditis, the mortality rate was 38.8% [29]. Although there are no randomized controlled clinical trials given the disease rarity; based on the above data and past experiences, we can at least extrapolate that early surgical intervention would be a reasonable approach especially in those with CVA related to S. lugdunensis IE.

## Conclusions

Compared to other organisms, S. lugdunensis seems to display a higher propensity to cause CVA in our review. In addition, embolic stroke secondary to S. lugdunensis IE appears to have a higher incidence in middle age men than women. Invasive procedures such as catheterization and vasectomy should be performed with proper sterile protocol to help reduce S. lugdunensis bacteremia or endocarditis. Although broad-spectrum antibiotics are typically the first medical treatment option, some patients may require a valve replacement surgery suggesting a higher virulence compared to other CoNS organism. The efficacy of medical therapy alone compared to the possible superiority of early surgical approach needs further investigation. However, it is challenging to conduct future large-scale studies to clarify the exact timeline of the surgical approach as S. lugdunensis endocarditis-related embolic stroke is a rare disease.
